# Supplementary material for: Comparing the signaling and transcriptome profiling landscapes of human iPSC-derived and primary rat neonatal cardiomyocytes
Source: Sci Rep. 2023 Jul 28;13:12248. doi: 10.1038/s41598-023-39525-4 (PMC10382583; doi:10.1038/s41598-023-39525-4)
Supplement: Supplementary file 1 — Supplementary Legends. [file 41598_2023_39525_MOESM1_ESM.pdf]

Supplemental Figure 1. Mean nuclear PKA activity over a 70-minute timeframe. Baseline normalized averaged nuclear PKA responses measured in **(A)** human iPSC-derived cardiomyocytes (hiPSC-CMs) and **(B)** rat neonatal cardiomyocytes (RNCMs). Grey shadow represents SEM.

Supplemental Figure 2. Graphical representations of PKA response clusters in hiPSC-CMs and RNCMs. Response clusters were plotted as a function of ExRai-AKAR2-NLS biosensor expression (RFU), ranging from **(A)** 0 – 5000, 1000 – 5000 and 1000 – 10 000. Biosensor expression did not appreciably influence the response clusters generated. Requesting the clustering algorithm to "find" four clusters instead of **(B)** three provided a better fit for the data.

Supplemental Figure 3. Bar plot representing drug diffusion patterns within different fields of imaged hiPSC-cardiomyocytes expressing ExRai-AKAR2-NLS. Charts display drug diffusion at the first timepoint post-stimulation with **(A)** norepinephrine (Welch's ANOVA p = 0.3260), **(B)** forskolin (Welch's ANOVA p = 0.0214), **(C)** isoproterenol (Welch's ANOVA = 0.0043, Dunnett's multiple comparisons test field 8 vs. 20 p = 0.0404), Error bars represent SEM.

Supplemental Figure 4. Single nuclei PKA activation patterns as observed in hiPSC-CM and RNCM cultures in response to angiotensinergic drugs. Heatmaps displaying nuclear PKA activity as measured in **(A)** hiPSC-CMs and **(B)** RNCMs. Single nuclear PKA data summarized as %ΔF/F (y-axis) as a function of time (x-axis). The data was partitioned into four clusters representing distinct nuclear behaviors, either exhibited sustained or transient responses to agonists while other nuclei failed to respond or experienced a decrease in activity compared to baseline. Representation of the four response clusters was plotted as a bar chart with percentages of nuclei belonging to each response cluster as observed in **(C)** hiPSC-CMs and **(D)** RNCMs. Experiments were performed using RNCMs isolated from 4 different neonatal rat pup litters and 3 independent cardiomyocyte differentiations from hiPSCs.

Supplemental Figure 5. Nuclear $ERK_{1/2}$ investigations in hiPSC-CM and RNCMs. Fluorescent microscopy image depicting expression of EKAREV-NLS donor in **(A)** hiPSC-CMs and **(B)** RNCMs. Agonist induced FRET responses plotted as a function of donor intensity in **(C)** hiPSC-CMs and RNCMs. Donor is expressed at higher intensities in hiPSC-CMs. **(D)** Averaged $ERK_{1/2}$ response profiles in both cardiomyocytes cell types. Grey shadow represents SEM.

Supplemental Figure 6. Graphical depiction of nuclear $ERK_{1/2}$ response clusters. The clustering algorithm was requested to partition data in either (A) three or (B) four clusters. Based on the clustering output, nuclei appeared to experience three response profiles as opposed to four. This is exemplified in panel (B) as two clusters, displayed with orange and plum colours, appear to overlap with one another. **(C)** Depiction of the sub-clustering applied on the 'Responder' population. It is apparent that the 'responding' nuclei can be further divided into two clusters, referred as low- and high- responders.

Supplemental Figure 7. Adrenergic ligand stimulation of $ERK_{1/2}$ action in hiPSC-CMS and RNCMs. Heatmaps displaying nuclear $ERK_{1/2}$ activity as measured in **(A)** hiPSC-CMs and **(B)** RNCMs. Single nuclear $ERK_{1/2}$ data summarized as %ΔF/F (y-axis- each line is a different cell) as a function of time (x-axis). The data was partitioned into three clusters representing distinct nuclear behaviors. CM nuclei either responded to the drug stimulations, resulting in $ERK_{1/2}$ activation, while others did not respond, and the last cluster of nuclei represented those that exhibited a decrease in $ERK_{1/2}$ activity compared to baseline.

Supplemental Figure 8. Single nuclei $ERK_{1/2}$ activation patterns as observed in hiPSC-CM and RNCM cultures in response to Ang II and β-arrestin biased peptides. Heatmaps displaying nuclear $ERK_{1/2}$ activity as measured in **(A)** hiPSC-CMs and **(B)** RNCMs. Single nuclear $ERK_{1/2}$ data summarized as $\%\Delta F/F$ (y-axis- each line is a different cell) as a function of time (x-axis). The data was partitioned into three clusters representing distinct nuclear behaviors. CM nuclei either responded to the drug stimulations, resulting in $ERK_{1/2}$ activation, while others did not respond, and the last cluster of nuclei represented those that exhibited a decrease in $ERK_{1/2}$ activity compared to baseline.

Supplemental Figure 9. Ang II results in $ERK_{1/2}$ activity in RNCMs. A) Representative western blot demonstrating that Ang II stimulation results in $ERK_{1/2}$ activity, depicted by pERK antibody at early timepoints, 5 minutes**. B)** Densitometry of three representative western blots. **C)** Full length blot of pERK data in (**A**). **D)** Full length blot of total ERK data in (**A**).

Supplemental Figure 10. Ligand independent RNA-seq based comparison of the endogenous signaling machinery expressed in RNCMs, hiPSC-CMs and HEK 293 cells. Exploratory analysis of gene sets associated with GPCR signal transduction investigating **(A)** class A GPCRs, **(B)** select cardiac relevant GPCRs, heterotrimeric G proteins **(C)** Gα, **(D)** Gβ and **(E)** Gγ as well as **(F)** effector expression profiles. Colorless heatmaps show transcript abundance measured in TPM, related to **Figure 5**. For comparison's sake, data from HEK 293 cells was included (published originally as Lukasheva et al., 2020 Sci Rep 10, 8779. https://doi.org/10.1038/s41598-020-65636-3).[20]

Supplemental Figure 11. Volcano plots demonstrating up- and down-regulated genes as determined by bulk RNAseq, 60 minutes after stimulation with various agonists.

Supplemental Figure 12. Ligand-independent comparative gene expression analysis in hiPSC-CMs and RNCMs. RNA-seq inferred transcript abundance measured as TPM of curated gene sets associated with **(A)** cardiac progenitor differentiation, **(B)** maturation-related genes.

Supplemental Figure 13. Transcript profiles of select energy metabolism-related gene sets. Transcript abundance (TPM) of gene involved in **(A)** glycolysis and **(B)** fatty acid metabolism.

Supplemental Figure 14. Exploration of get sets associated with cardiomyocyte behavior and function. RNA-seq inferred transcript abundance of genes related with **(A)** cardiac muscle contraction **(B)** regulation of heart rate by cardiac conduction and **(C)** ion channels that generate cardiac action potential. Colorless heatmaps show transcript abundance measured in TPM, related to **Figure 6**.
