# Supplementary material for: Comparing the signaling and transcriptome profiling landscapes of human iPSC-derived and primary rat neonatal cardiomyocytes
Source: Sci Rep. 2023 Jul 28;13:12248. doi: 10.1038/s41598-023-39525-4 (PMC10382583; doi:10.1038/s41598-023-39525-4)
Supplement: Supplementary file 2 — Supplementary Figures. [file 41598_2023_39525_MOESM2_ESM.pdf]

Supplemental Figure 1

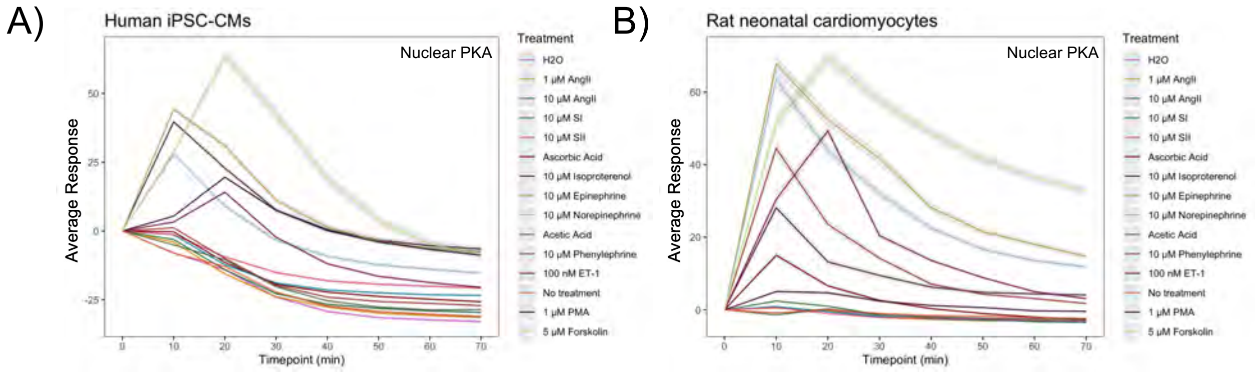

Supplemental Figure 2

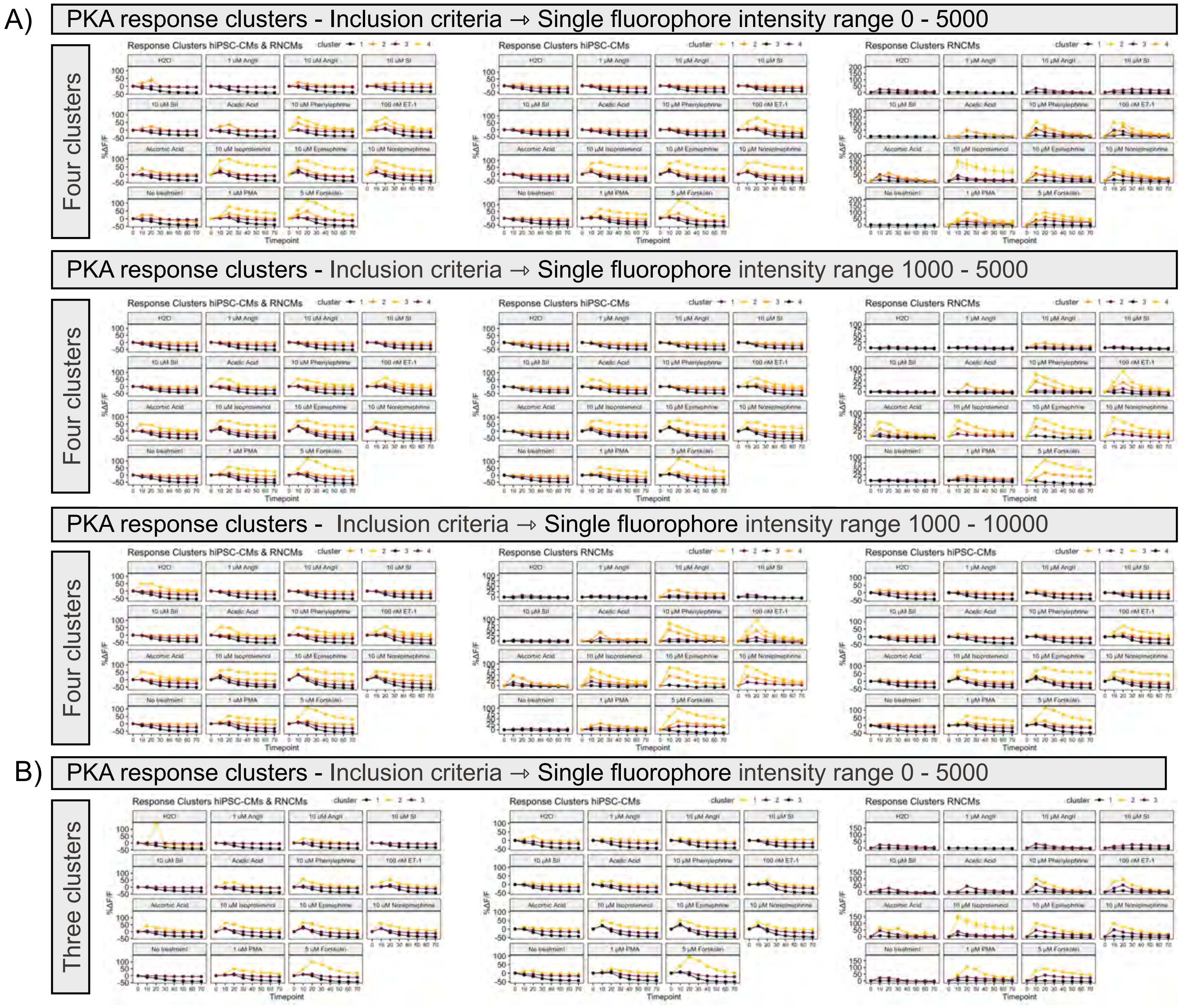

Supplemental Figure 3

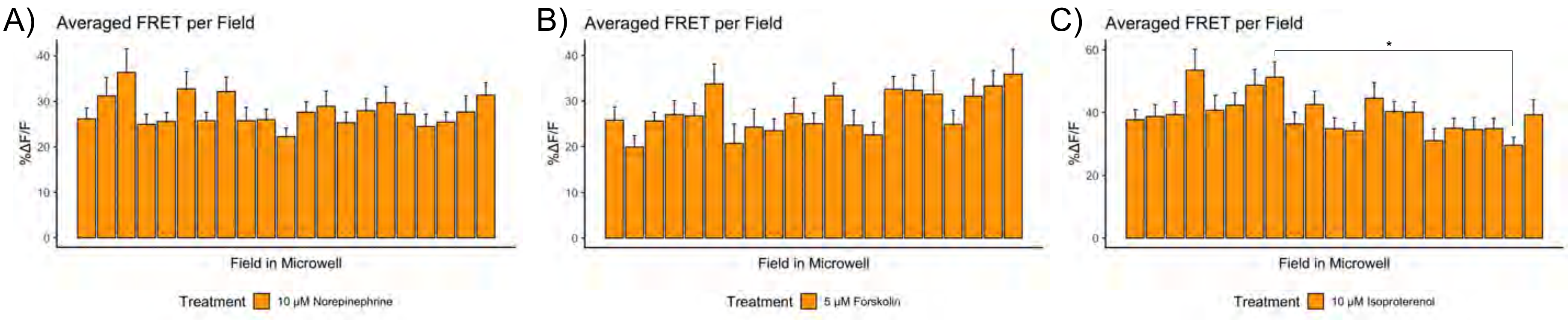

Supplemental Figure 4

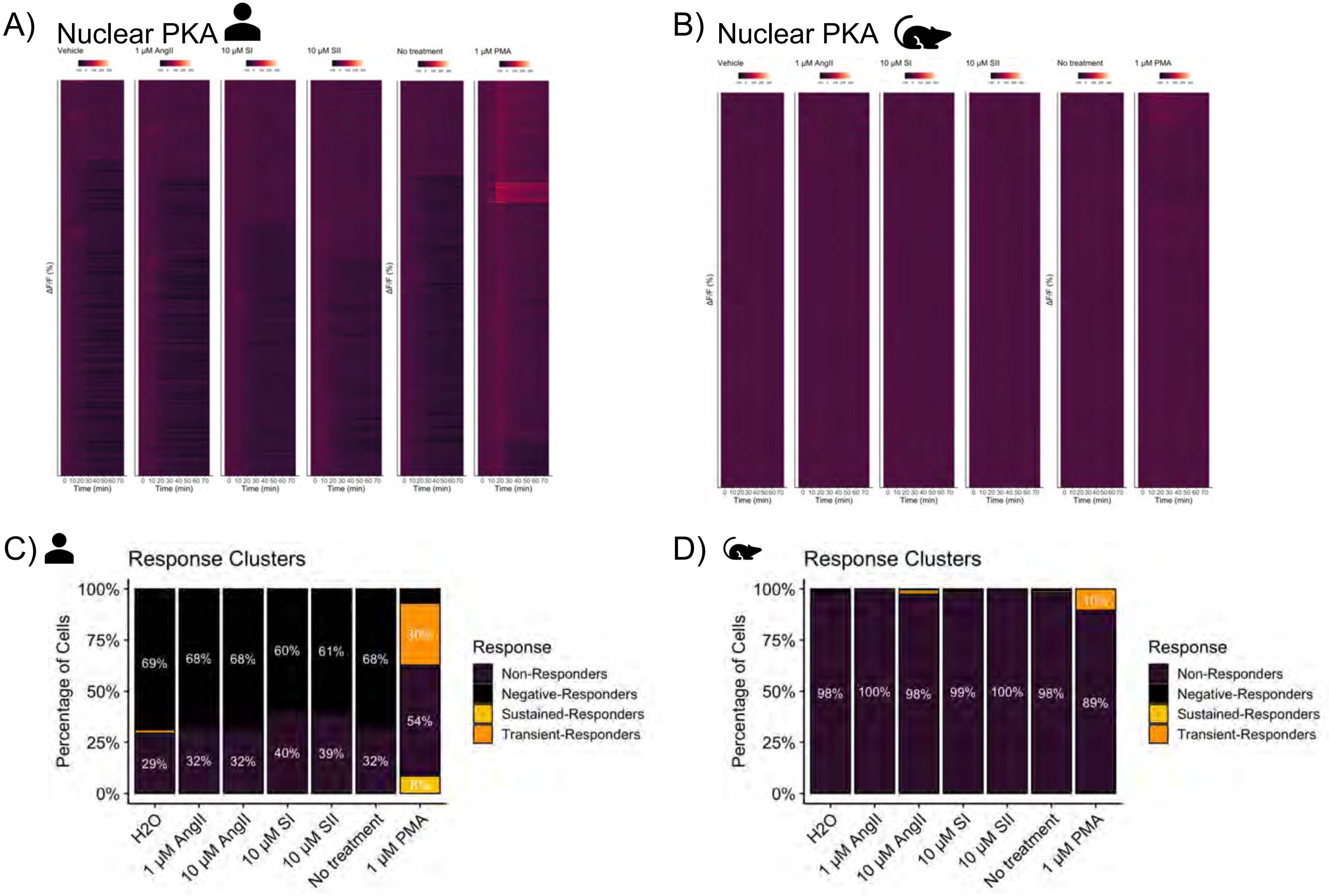

Supplemental Figure 5

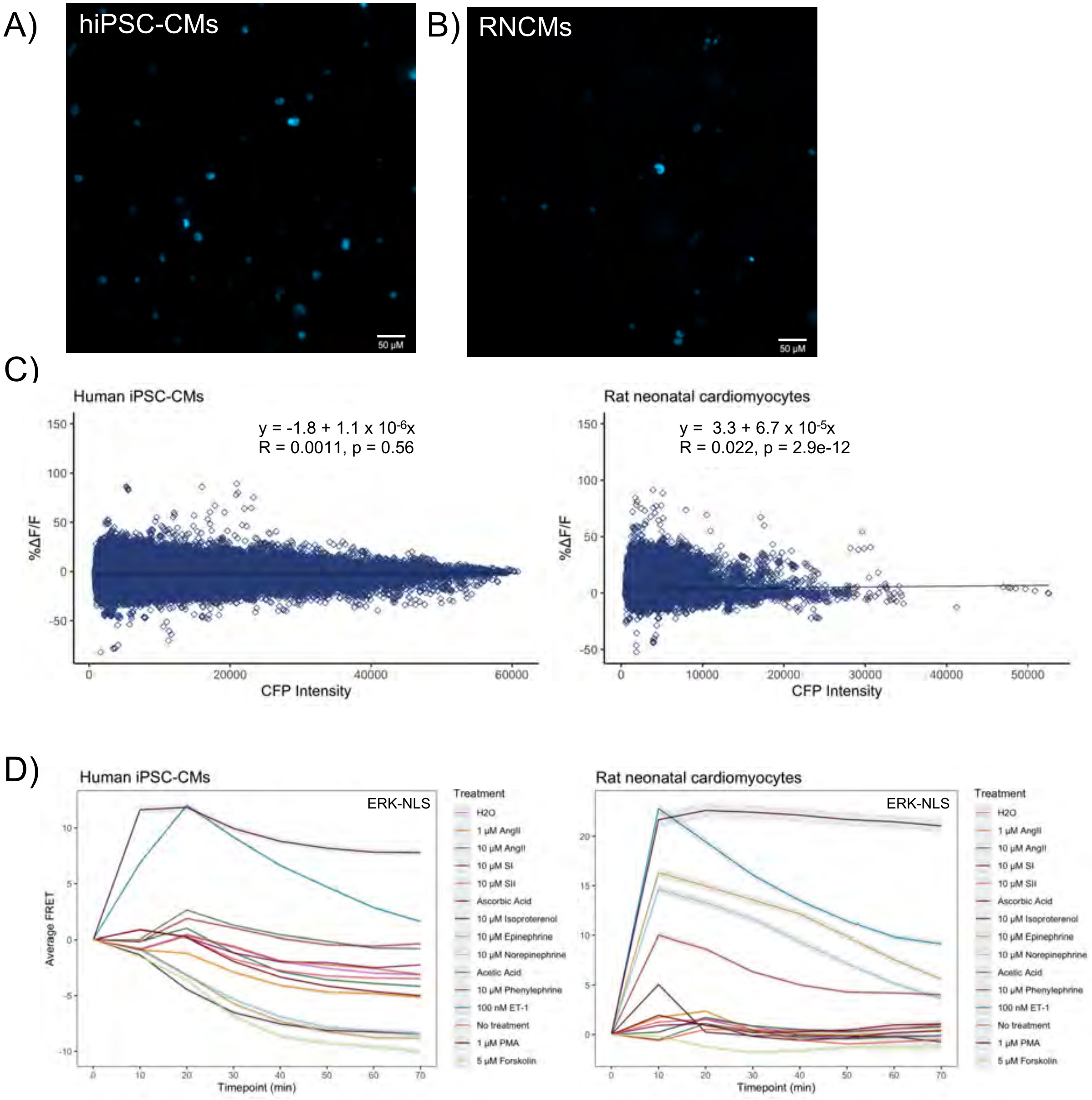

Supplemental Figure 6

A) ERK response clusters - Inclusion criteria → CFP intensity range 2000 - 5000

Three clusters

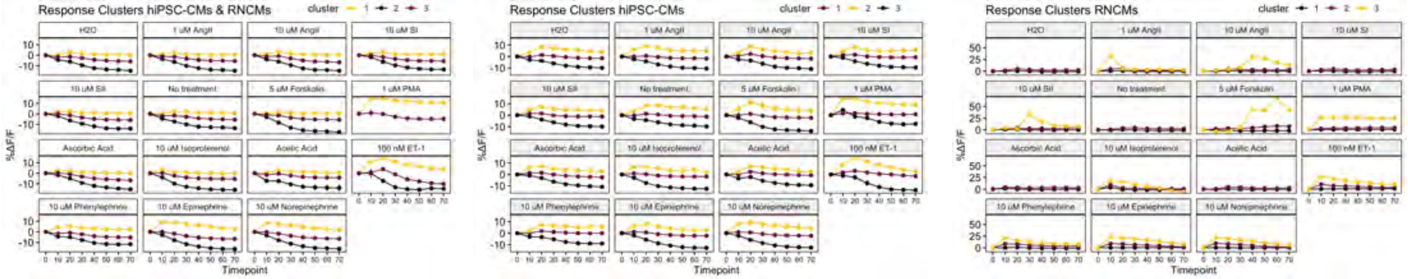

B) ERK response clusters - Inclusion criteria → CFP intensity range 2000 - 5000

Four clusters

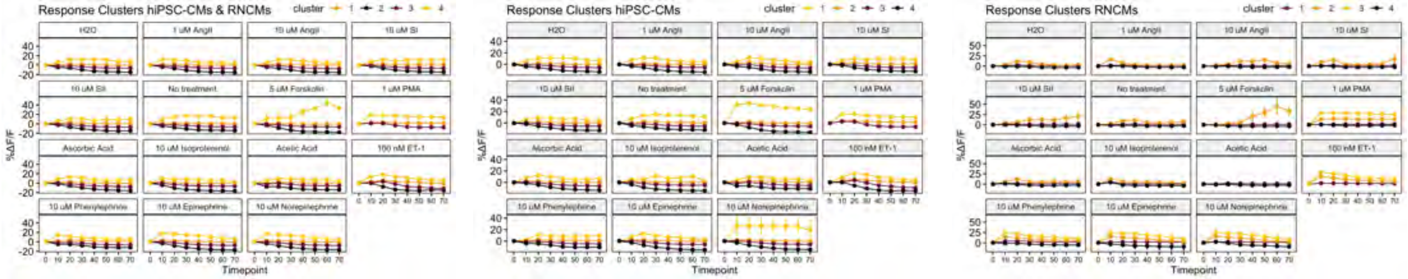

C) Responders

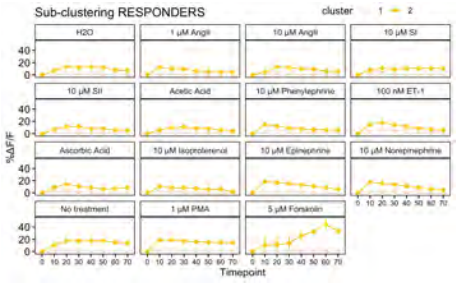

Supplemental Figure 7

A) Nuclear ERK

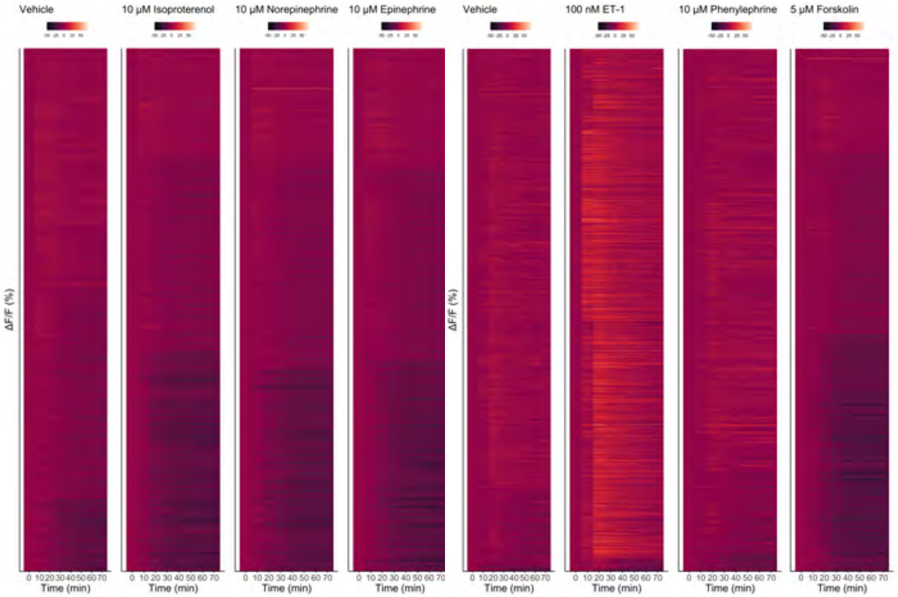

B) Nuclear ERK

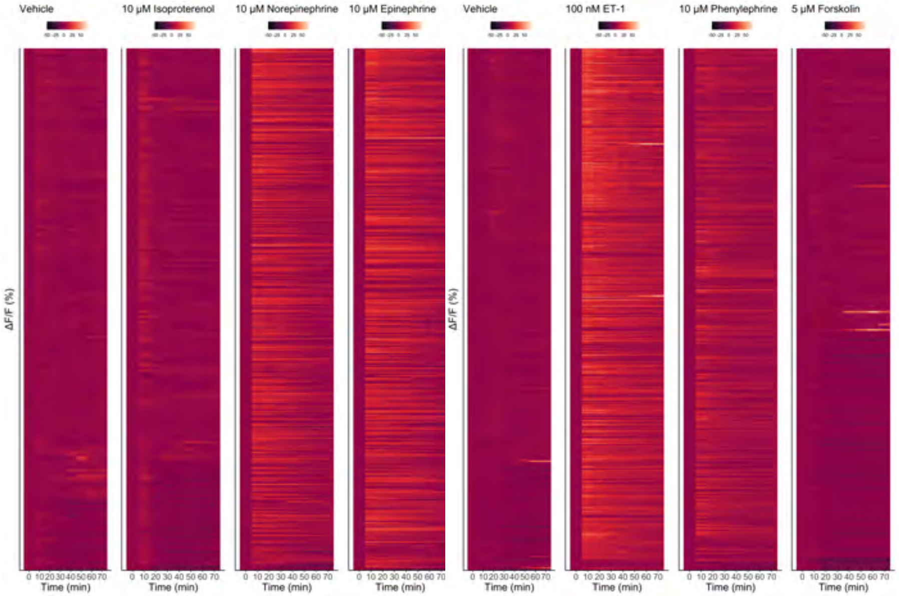

Supplemental Figure 8

A) Nuclear ERK

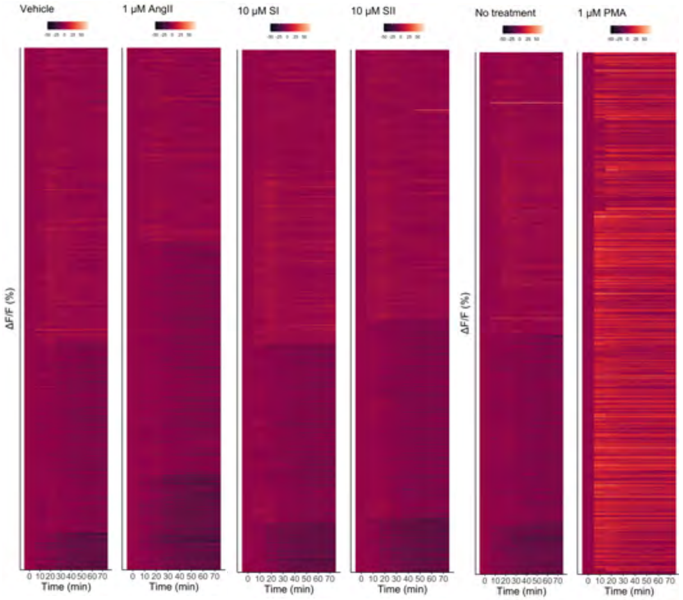

B) Nuclear ERK

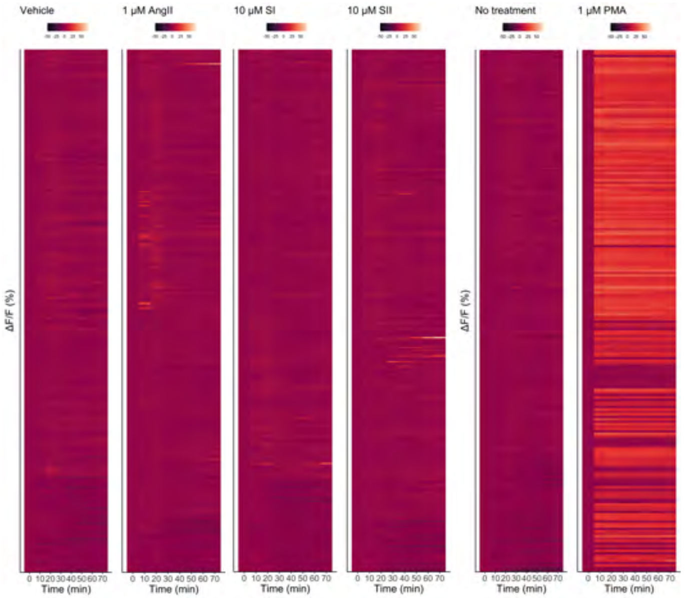

Supplemental Figure 9

A) RNCMs - 1 μM Ang II

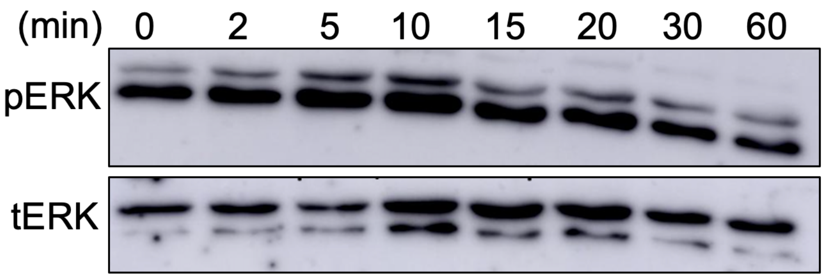

B)

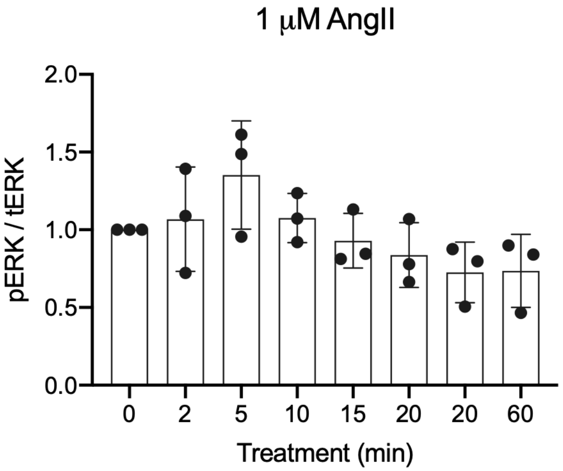

C)

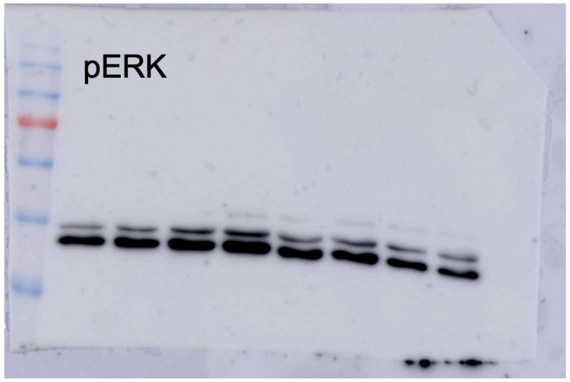

D)

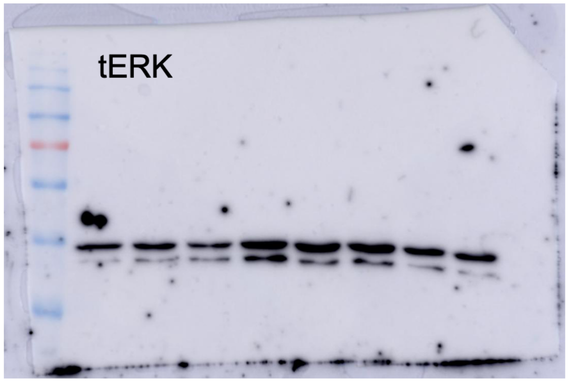

Supplemental Figure 10

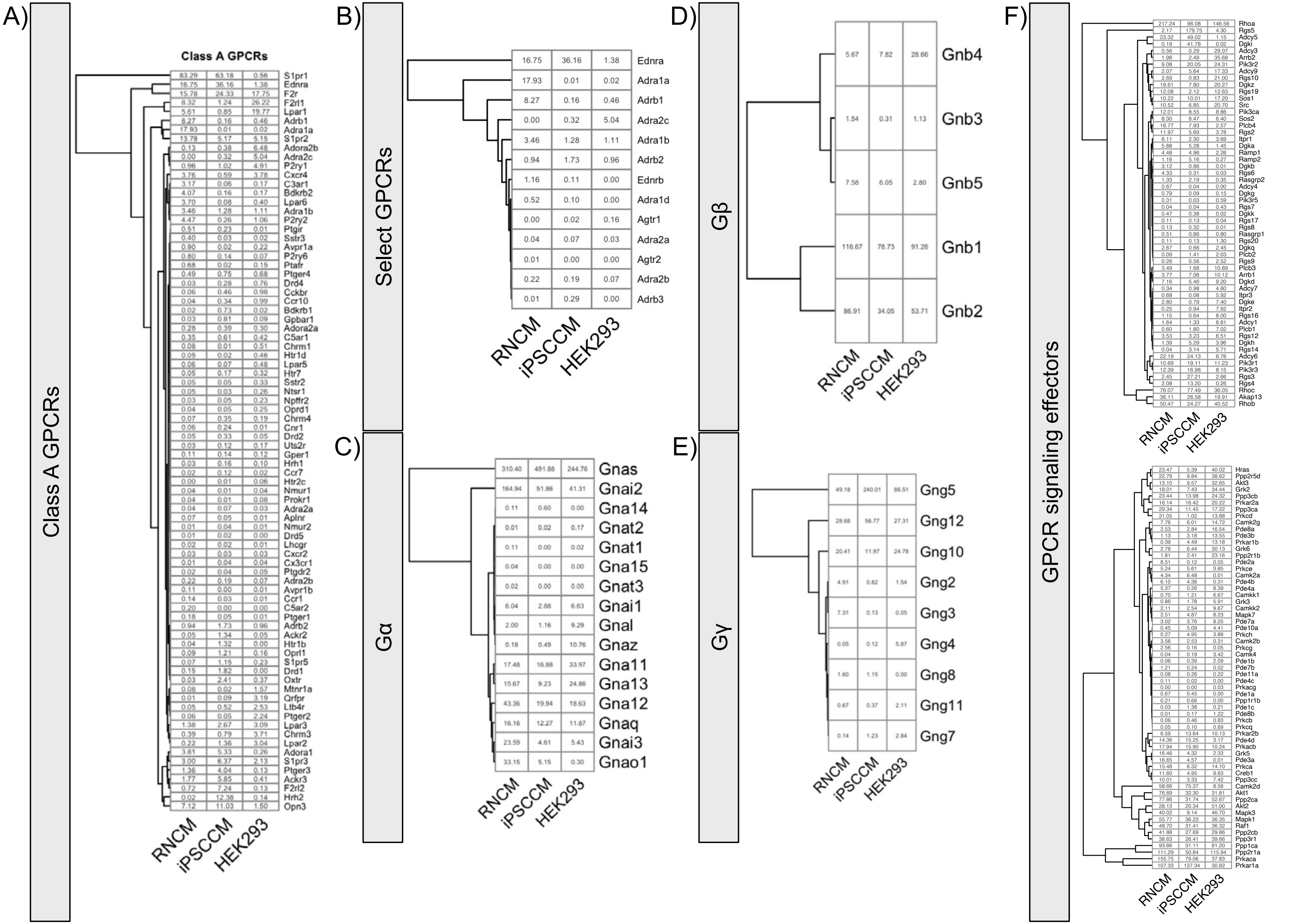

Supplemental Figure 11

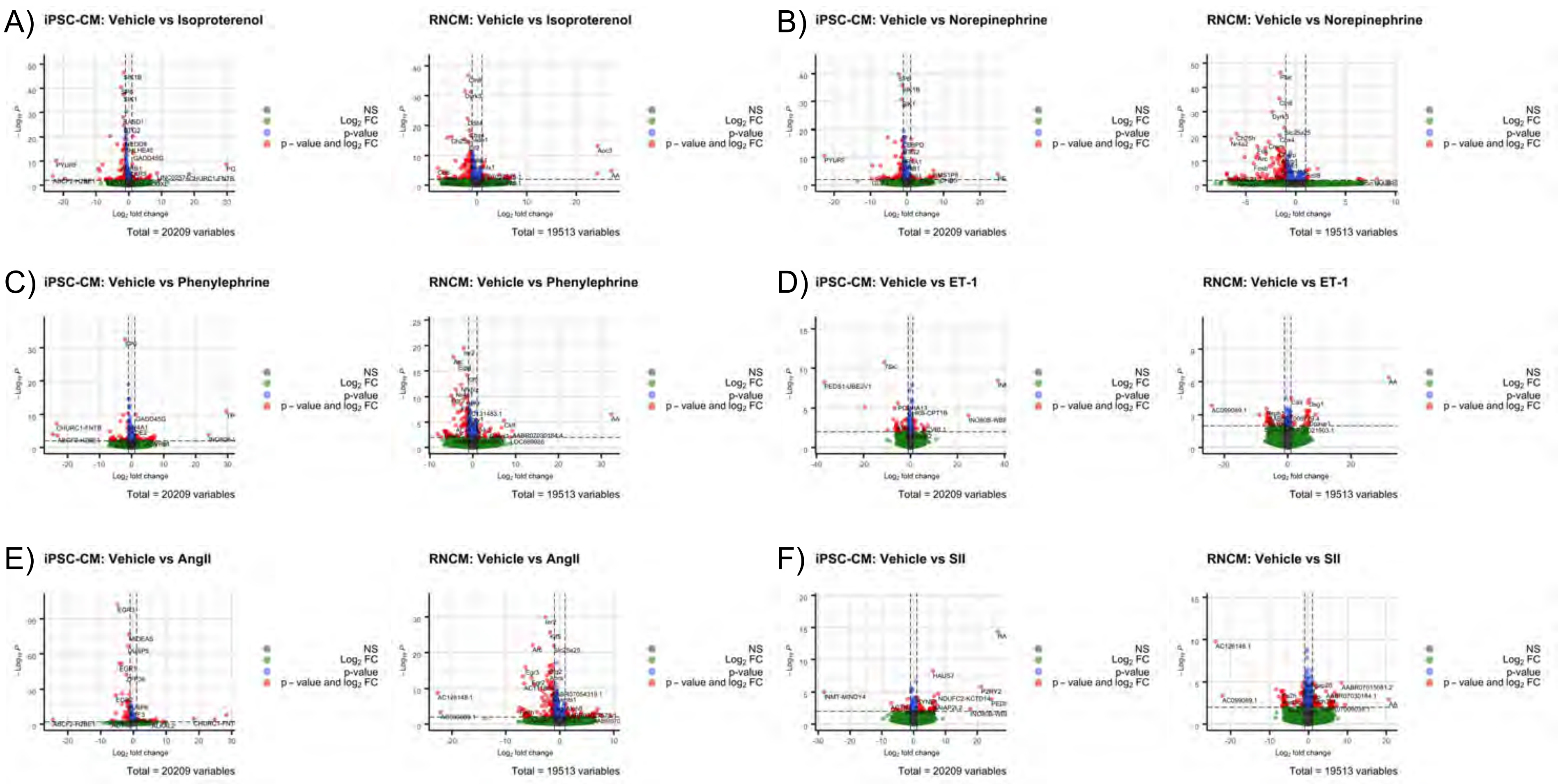

Supplemental Figure 12

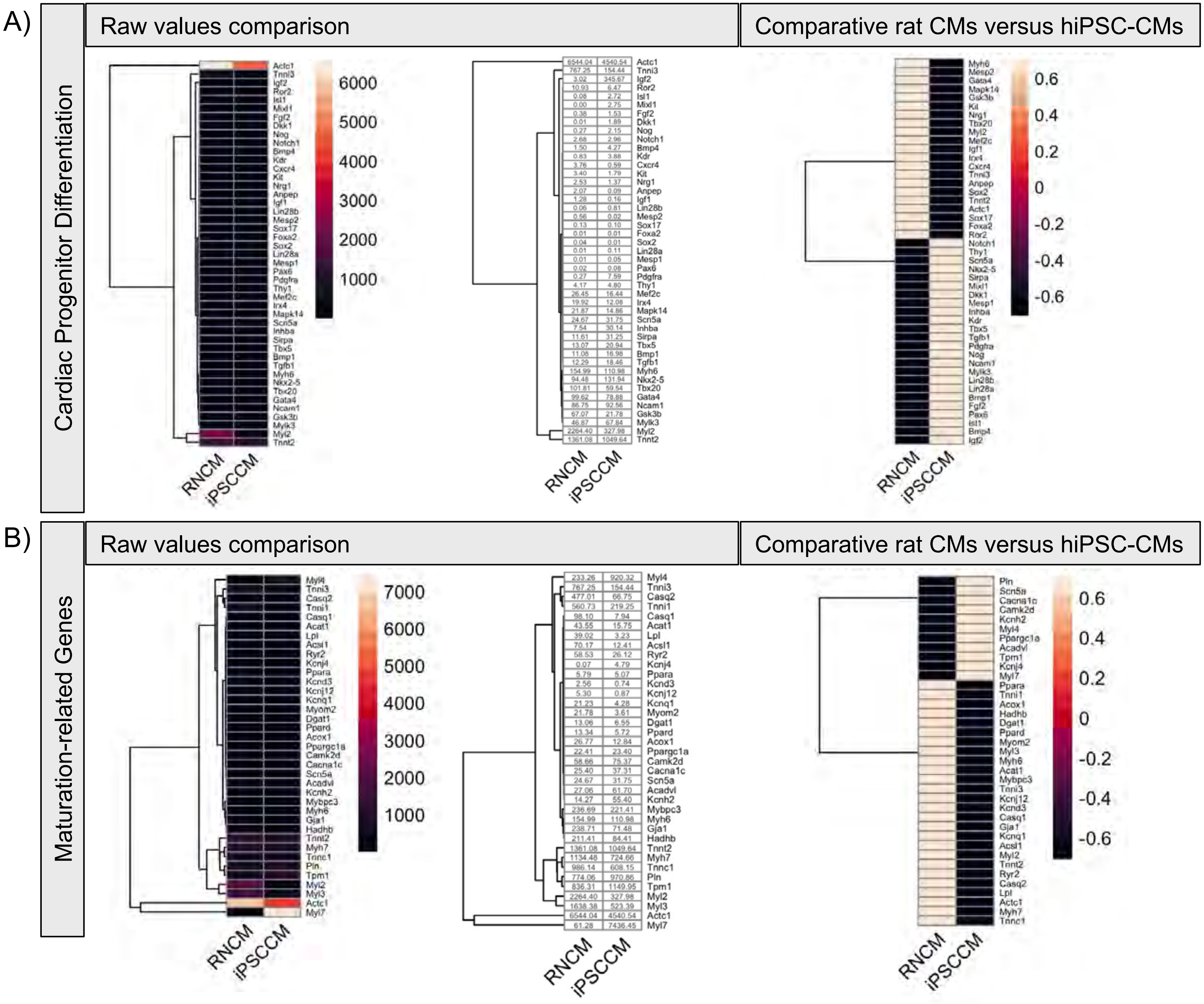

Supplemental Figure 13

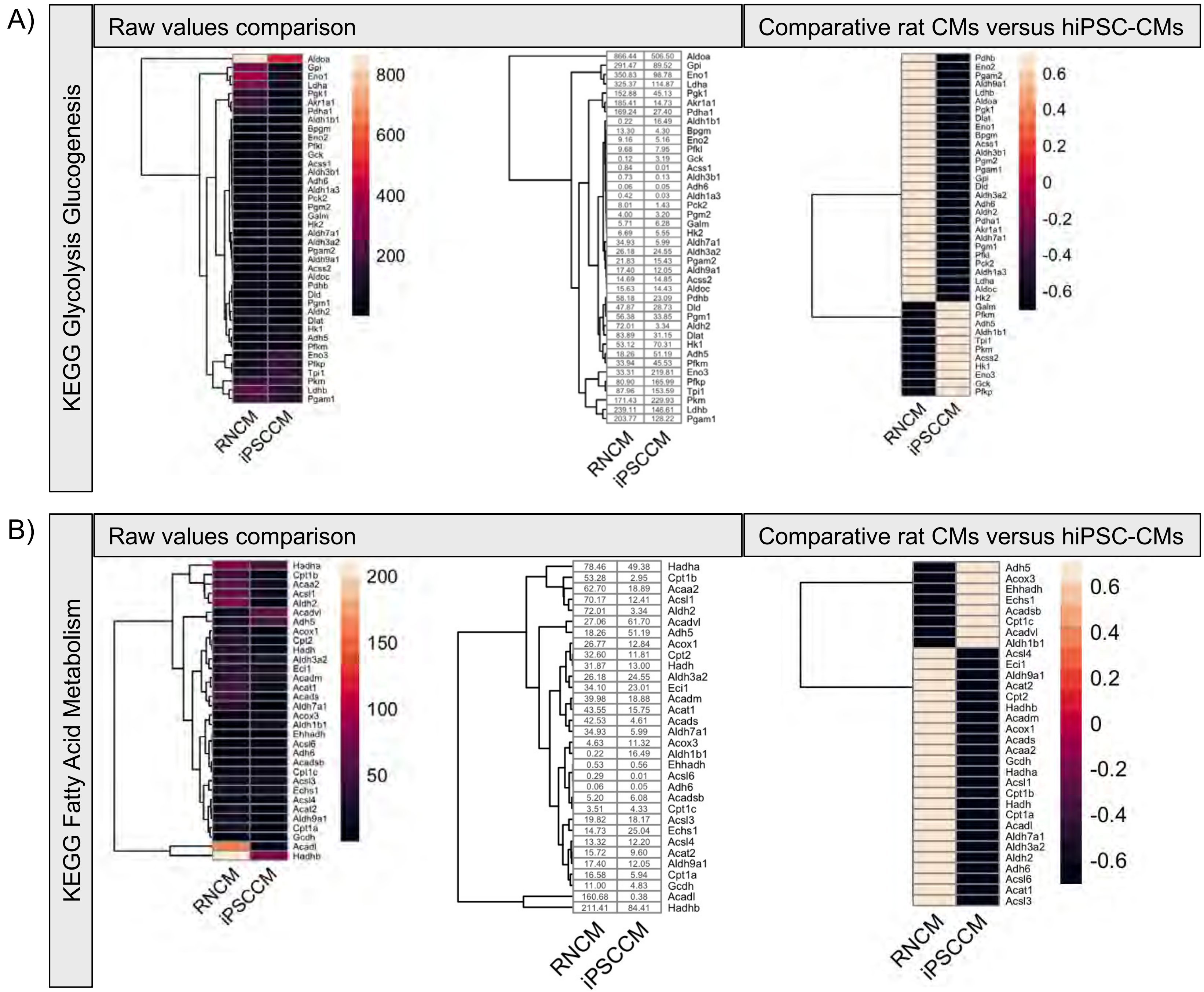

Supplemental Figure 14

A)

Cardiac Muscle Contraction

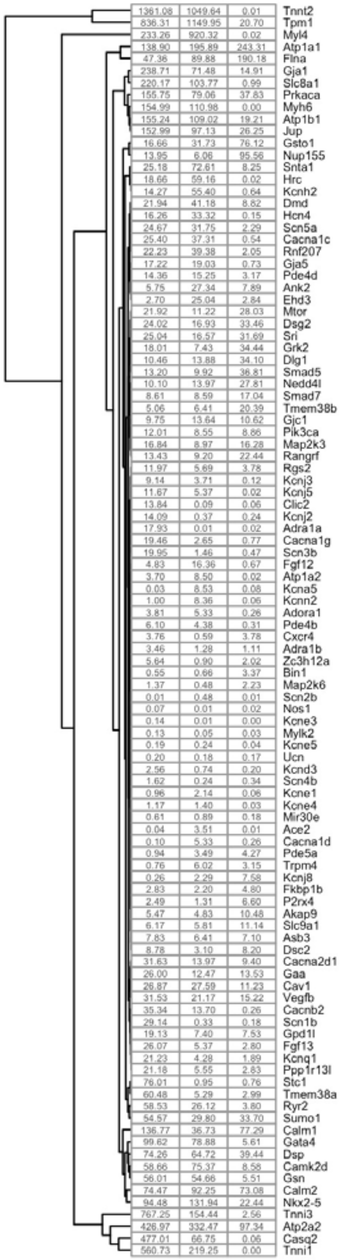

RNCM	iPSCCM	HEK293	
1361.08	1049.64	0.01	Tnnt2
836.31	1149.95	20.70	Tpm1
233.26	920.32	0.02	Myl4
138.90	195.89	243.31	Atp1a1
47.36	89.88	190.18	Flna
238.71	71.48	14.91	Gja1
220.17	103.77	0.99	Slc8a1
155.75	79.06	37.83	Prkaca
154.99	110.98	0.00	Myh6
155.24	109.02	19.21	Atp1b1
152.99	97.13	26.25	Jup
16.66	31.73	76.12	Gsto1
13.95	6.06	95.56	Nup155
25.18	72.61	8.25	Snta1
18.66	59.16	0.02	Hrc
14.27	55.40	0.64	Kcnh2
21.94	41.18	8.82	Dmd
16.26	33.32	0.15	Hcn4
24.67	31.75	2.29	Scn5a
25.40	37.31	0.54	Cacna1c
22.23	39.38	2.05	Rnf207
17.22	19.03	0.73	Gja5
14.36	15.25	3.17	Pde4d
5.75	27.34	7.89	Ank2
2.70	25.04	2.84	Ehd3
21.92	11.22	28.03	Mtor
24.02	16.93	33.46	Dsg2
25.04	16.57	31.69	Sri
18.01	7.43	34.44	Grk2
10.46	13.88	34.10	Dlg1
13.20	9.92	36.81	Smad5
10.10	13.97	27.81	Nedd4l
8.61	8.59	17.04	Smad7
5.06	6.41	20.39	Tmem38b
9.75	13.64	10.62	Gjc1
12.01	8.55	8.86	Pik3ca
16.84	8.97	16.28	Map2k3
13.43	9.20	22.44	Rangrf
11.97	5.69	3.78	Rgs2
9.14	3.71	0.12	Kcnj3
11.67	5.37	0.02	Kcnj5
13.84	0.09	0.06	Clic2
14.09	0.37	0.24	Kcnj2
17.93	0.01	0.02	Adra1a
19.46	2.65	0.77	Cacna1g
19.95	1.46	0.47	Scn3b
4.83	16.36	0.67	Fgf12
3.70	8.50	0.02	Atp1a2
0.03	8.53	0.08	Kcna5
1.00	8.36	0.06	Kcnn2
3.81	5.33	0.26	Adora1
6.10	4.38	0.31	Pde4b
3.76	0.59	3.78	Cxcr4
3.46	1.28	1.11	Adra1b
5.64	0.90	2.02	Zc3h12a
0.55	0.66	3.37	Bin1
1.37	0.48	2.23	Map2k6
0.01	0.48	0.01	Scn2b
0.07	0.01	0.02	Nos1
0.14	0.01	0.00	Kcne3
0.13	0.05	0.03	Mylk2
0.19	0.24	0.04	Kcne5
0.20	0.18	0.17	Ucn
2.56	0.74	0.20	Kcnd3
1.62	0.24	0.34	Scn4b
0.96	2.14	0.06	Kcne1
1.17	1.40	0.03	Kcne4
0.61	0.89	0.18	Mir30e
0.04	3.51	0.01	Ace2
0.10	5.33	0.26	Cacna1d
0.94	3.49	4.27	Pde5a
0.76	6.02	3.15	Trpm4
0.26	2.29	7.58	Kcnj8
2.83	2.20	4.80	Fkbp1b
2.49	1.31	6.60	P2rx4
5.47	4.83	10.48	Akap9
6.17	5.81	11.14	Slc9a1
7.83	6.41	7.10	Asb3
8.78	3.10	8.20	Dsc2
31.63	13.97	9.40	Cacna2d1
26.00	12.47	13.53	Gaa
26.87	27.59	11.23	Cav1
31.53	21.17	15.22	Vegfb
35.34	13.70	0.26	Cacnb2
29.14	0.33	0.18	Scn1b
19.13	7.40	7.53	Gpd1l
26.07	5.37	2.80	Fgf13
21.23	4.28	1.89	Kcnq1
21.18	5.55	2.83	Ppp1r13l
76.01	0.95	0.76	Stc1
60.48	5.29	2.99	Tmem38a
58.53	26.12	3.80	Ryr2
54.57	29.80	33.70	Sumo1
136.77	36.73	77.29	Calm1
99.62	78.88	5.61	Gata4
74.26	64.72	39.44	Dsp
58.66	75.37	8.58	Camk2d
56.01	54.66	5.51	Gsn
74.47	92.25	73.08	Calm2
94.48	131.94	22.44	Nkx2-5
767.25	154.44	2.56	Tnni3
426.97	332.47	97.34	Atp2a2
477.01	66.75	0.06	Casq2
560.73	219.25	0.00	Tnni1

B)

Regulation HR by Cardiac Conduction

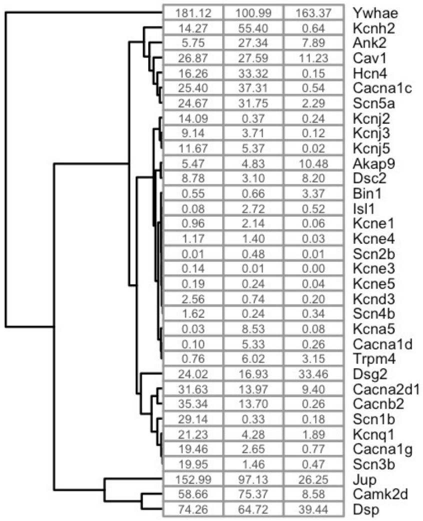

RNCM	iPSCCM	HEK293	
181.12	100.99	163.37	Ywhae
14.27	55.40	0.64	Kcnh2
5.75	27.34	7.89	Ank2
26.87	27.59	11.23	Cav1
16.26	33.32	0.15	Hcn4
25.40	37.31	0.54	Cacna1c
24.67	31.75	2.29	Scn5a
14.09	0.37	0.24	Kcnj2
9.14	3.71	0.12	Kcnj3
11.67	5.37	0.02	Kcnj5
5.47	4.83	10.48	Akap9
8.78	3.10	8.20	Dsc2
0.55	0.66	3.37	Bin1
0.08	2.72	0.52	Isl1
0.96	2.14	0.06	Kcne1
1.17	1.40	0.03	Kcne4
0.01	0.48	0.01	Scn2b
0.14	0.01	0.00	Kcne3
0.19	0.24	0.04	Kcne5
2.56	0.74	0.20	Kcnd3
1.62	0.24	0.34	Scn4b
0.03	8.53	0.08	Kcna5
0.10	5.33	0.26	Cacna1d
0.76	6.02	3.15	Trpm4
24.02	16.93	33.46	Dsg2
31.63	13.97	9.40	Cacna2d1
35.34	13.70	0.26	Cacnb2
29.14	0.33	0.18	Scn1b
21.23	4.28	1.89	Kcnq1
19.46	2.65	0.77	Cacna1g
19.95	1.46	0.47	Scn3b
152.99	97.13	26.25	Jup
58.66	75.37	8.58	Camk2d
74.26	64.72	39.44	Dsp

C)

Ion channels that generate AP

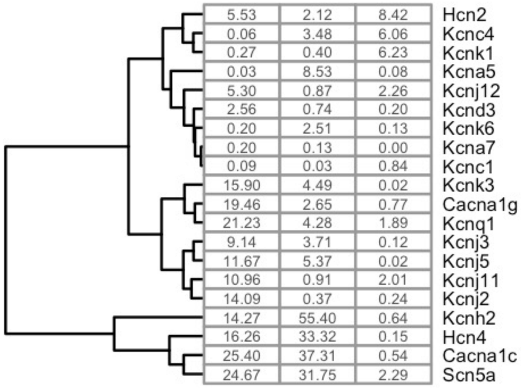

RNCM	iPSCCM	HEK293	
5.53	2.12	8.42	Hcn2
0.06	3.48	6.06	Kcnc4
0.27	0.40	6.23	Kcnk1
0.03	8.53	0.08	Kcna5
5.30	0.87	2.26	Kcnj12
2.56	0.74	0.20	Kcnd3
0.20	2.51	0.13	Kcnk6
0.20	0.13	0.00	Kcna7
0.09	0.03	0.84	Kcnc1
15.90	4.49	0.02	Kcnk3
19.46	2.65	0.77	Cacna1g
21.23	4.28	1.89	Kcnq1
9.14	3.71	0.12	Kcnj3
11.67	5.37	0.02	Kcnj5
10.96	0.91	2.01	Kcnj11
14.09	0.37	0.24	Kcnj2
14.27	55.40	0.64	Kcnh2
16.26	33.32	0.15	Hcn4
25.40	37.31	0.54	Cacna1c
24.67	31.75	2.29	Scn5a
